# Congenital laryngeal saccular cyst

**DOI:** 10.1590/S1808-86942012000300025

**Published:** 2015-10-14

**Authors:** Luiz Ubirajara Sennes, Rui Imamura, Ronaldo Frizzarini, Adriana Hachiya, Azis Arruda Chagury

**Affiliations:** a Professor of Otorhinolaryngology in the Medical School of the University of São Paulo.; b Assisting Physician-PhD in the Division of Clinical ENT at the Hospital das Clínicas of the Medical School of the University of São Paulo.; c Assisting Physician-PhD in the Division of Clinical ENT at the Hospital das Clínicas of the Medical School of the University of São Paulo.; d Assisting Physician-PhD in the Division of Clinical ENT at the Hospital das Clínicas of the Medical School of the University of São Paulo.; e MD, Specializaton Student on ENT in the Division of Clinical ENT at the Hospital das Clínicas of the Medical School of the University of São Paulo. Divisão de Clínica Otorrinolaringológica e no Departamento de Radiologia do Hospital das Clínicas da Faculdade de Medicina da Universidade de São Paulo.

**Keywords:** cysts, infant newborn, laryngeal mucosa, larynx

## INTRODUCTION

Laryngeal saccular cysts account for a rare benign disease caused by atresia of the orifice of the laryngeal saccule or by retention of mucus from the ventricle's submucosal glands. It may be congenital or acquired. Acquired cases may have their origins in prolonged orotracheal intubation or laryngeal surgery^1^.

Laryngeal saccular cysts are among the causes of laryngeal stridor in neonates and make up the differential diagnosis roster against laryngomalacia, vocal fold paralysis, congenital subglottic stenosis, laryngeal web, and laryngocele^2^. It may severely compromise airways. However, some 50% of the cases are asymptomatic and are found only during autopsy^3^.

This case report aims to show the relevance of saccular cysts in the differential diagnosis of neonates with laryngeal stridor, describe the treatment and the evolution of the patient.

## CASE PRESENTATION

The patient is a full-term neonate, born from e Cesarean section, presenting sings of distress and meconium aspiration (Apgar 6, 8 and 9). Right after birth the patient showed signs of respiratory discomfort and meconium aspiration, and was intubated. Chest X-rays, however, ruled out such diagnostic possibility and the patient was extubated. The neonate still presented stridor during inhalation and showed sings of discomfort in times of physical strain, mainly as he cried and was fed. Nasal endoscopy showed a cyst growing towards the aryepiglottic fold consistent with a saccular cyst.

At 48 hours of age the patient underwent a relief cyst marsupialization procedure. He improved from the discomfort, but the laryngeal stridor was still present, supposedly due to postoperative edema. One week later the patient's discomfort had grown worse. Nasal endoscopy showed edema organization and a likely recurrence of the cyst. He was sent to surgery.

**Figure 1:**
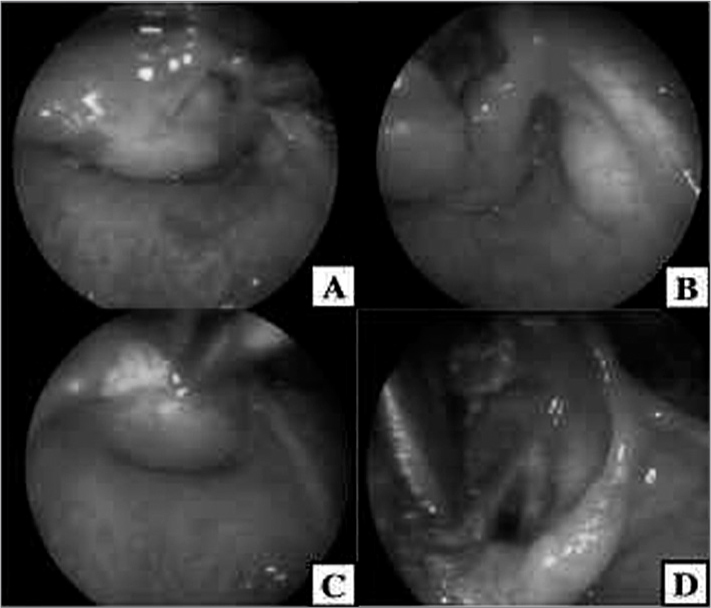
A. Saccular cyst completely obstructing the laryngeal lumen. B- Glottis is exposed as the cyst is moved away. C- Cyst cauterization. D- Glottis is exposed as the cyst is resected.

The patient could not be intubated during the procedure. A tracheostomy was carried out to offer him a viable airway and better expose the cyst. The endolaryngeal approach was chosen to remove the upper portion of the cyst and the cyst was then marsupialized (Figure 1). Pathology tests showed fragments of fibromuscular tissue and serous/mucous glands covered with non-keratinized squamous ciliated pseudostratified columnar epithelium.

The child progressed well after surgery, and the tracheostomy cannula was removed seven days into postoperative care. Nasal endoscopic examination done thirty days after surgery showed no signs of cyst recurrence.

## DISCUSSION

Although there are similarities between saccular cysts and laryngocele, cysts do not contain air in them and do not connect to the laryngeal lumen, as is the case with laryngocele. Cysts may cause dyspnea, dysphagia, and laryngeal stridor. Patients need to be examined endoscopically and additionally by imaging if a firm diagnosis cannot be reached.

The classical treatment for saccular cysts is endoscopic surgery. In 1978, Hollinger et al.^4^ proposed that the cyst contents should be removed by suction. Given the high rates of cyst recurrence, some authors advocate cyst marsupialization as the treatment of choice. In this procedure the cyst is dissected to its base, in the orifice of the saccule, and is then amputated, as done in our patient's second procedure.

Less invasive approaches may also be used, such as the endoscopic extended ventriculotomy, thus sparing the patient from a tracheostomy and open surgery, aside from diminishing morbidity and mortality rates^5^.

Another option is the external approach, to be done after the cyst has relapsed after one or two attempts to remove it endoscopically, when there are extra-laryngeal components, or more extensive cysts. Albeit less conservative, the external approach presents lower recurrence rates when well indicated, reduces the course of the disease, and prevents postoperative complications.

## CONCLUSION

The ENT examination of neonates with laryngeal stridor must be thorough and detailed. Patient history and physical examination are utterly important and include endoscopic examination to rule out nasal and laryngeal diseases. Although saccular cysts are rare, early diagnosis allows for definitive treatment, improvement from respiratory discomfort, and precludes the need for prolonged intubation and all potential complications associated with it.
